# A Case Report of Doege–Potter Syndrome: A Rare Cause of Hypoglycemia in a Patient without Diabetes

**DOI:** 10.3390/jcm12123984

**Published:** 2023-06-12

**Authors:** Chiara Corsano, Matteo Paradiso, Ester Daniela Laudadio, Francesco Sollitto, Olga Lamacchia

**Affiliations:** 1 Endocrinology Unit, Department of Medical and Surgical Sciences, University of Foggia, 71122 Foggia, Italy; chiara.corsano@unifg.it (C.C.); matteoparadiso@gmail.com (M.P.); esterdaniela@virgilio.it (E.D.L.); 2 Institute of Thoracic Surgery, Department of Medical and Surgical Sciences, University of Foggia, 71122 Foggia, Italy; francesco.sollitto@unifg.it

**Keywords:** paraneoplastic hypoglycemia, Doege-Potter syndrome, big-IGF-2, NICTH, case report

## Abstract

Hypoglycemia in patients without diabetes is a diagnostic challenge for the endocrinologist. Sometimes it is related to rare causes such as Doege–Potter Syndrome (DPS). DPS is caused by an abnormal insulin-like grow factor 2(IGF-2) that retains part of the E domain during the production process, resulting in a longer peptide called “big-IGF-2”. We present a case report of DPS with emphasis on the diagnosis and especially on the difficulties in interpreting the biochemical findings. An elderly patient with an intrathoracic neoplasm and hypoglycemia underwent various tests: insulin autoantibodies and fasting test were both negative. She had low values of IGF-1 and normal values of IGF-2 that apparently excludes a diagnosis of DPS. The evaluation of the IGF-2/IGF-1 ratio is the most important test because a ratio >10 is widely considered to be indicative of non-islet cell tumor hypoglycemia (NICTH). Glucose infusion and steroid therapy were used to control the hypoglycemia, but the definitive treatment was surgery, which almost immediately reversed the hypoglycemia. The differential diagnosis of hypoglycemia should include rare causes such as DPS, and the IGF-2/IGF-1 ratio is a useful tool.

## 1. Introduction

Hypoglycemia is unusual in people without diabetes. The causes to be considered are different in ill or medicated individuals (drugs, critical illness, hormone deficiency, NICTH) and in apparently well individuals (endogenous hyperinsulinism, accidental, surreptitious or malicious hypoglycemia) [1].

NICTH is a rare syndrome caused by the production of an insulin-like factor that leads to hypoglycemia. NICTH is known as DPS when associated with a solitary fibrous tumor (SFT). STF is a tumor of mesenchymal origin which is associated with DPS in less than 5% of cases [2,3,4].

NICTH is associated with impaired post-translational processing of IGF-2. IGF-2 is initially synthesized as pre-pro-IGF-2. In the post-translational process the first phase consists of the cleavage of the signal peptide with the formation of pro-IGF-2. Pro-IGF-2 is first glycosylated in the E domain, then cleaved by a convertase to form mature IGF-2. In NICTH the convertase does not completely cut the E-domain resulting in a longer peptide called “big-IGF-2” [5]. IGF-2 and big-IGF-2 can interact with several receptors: IGF receptor 1, IGF receptor 2, insulin receptor type A and B and hybrid receptors that bind both IGF and insulin with several affinities. IGF binding proteins (IGFBPs) permit the plasma transport of IGFs. IGFBPs form both dimeric complexes (IGFBP + IGF) and ternary complexes (IGFBP + IGF + labile acid subunit (ALS)). The latter, formed by IGFBP-3 or IGFBP-5, are more stable and larger and less prone to release the substrate to the tissues [6,7]. In NICTH, the modification of the IGF-2/big-IGF-2 ratio determines a pronounced formation of dimeric complexes which have enhanced bioavailability because they easily cross capillary membranes to interact with receptors [8,9]. The different expression of the receptors in tissues drives the peripheral effects of increased big-IGF-2 and IGF-2. In the pituitary gland, GH secretion is reduced. A lower level of GH acts in the liver by reducing the secretion of IGFBP-3, ALS, IGF-1. The presence of lower values of IGFBP-3 causes the formation of dimeric complexes and thus a higher availability of IGF-2/big-IGF-2 favoring a vicious cycle. In the pancreas and in adipose tissue, the result is a reduction in both glucagon secretion and lipolysis. A greater glucose uptake in muscles generates hypoglycemia [10]. We present a case report of DPS with emphasis on the diagnosis and especially on difficulties in interpreting the biochemical findings. The purpose of this case report is therefore two-fold: to highlight the diagnostic difficulties of DPS, but also to show a potential type of therapeutic approach in a context where there are no randomized clinical trials to suggest a more appropriate treatment.

## 2. Case Report

We present a case of persistent hypoglycemia in a 86-year-old woman. Our patient was affected by thyroid goiter, hypertension, dyslipidemia, osteoporosis, essential tremor, and dementia.

Two months earlier, the patient had undergone a chest computerized tomography (CT) scan, which revealed a large tumor-like lung formation (12 cm) in close relation to the parietal pleura; the result of a recent biopsy consisted of bronchial mucosal fragment showing only chronic inflammation.

The patient presented to the Emergency Department with motor instability. Blood glucose was 30 mg/dL. The patient was immediately treated with 33% glucose infusion with resolution of symptoms. During hospitalization, the patient experienced multiple episodes of hypoglycemia. In order to determine the cause of the hypoglycemia, a careful medical history was taken—which excluded pharmacological causes—and several blood tests were performed—with the only evidence of hypokalemia (3 mEq/L). Negative insulin autoantibodies ruled out Hirata’s Disease. Plasma cortisol level was measured at 8 A.M. in the morning (13.13 μg/mL) to exclude adrenal insufficiency. On the second day of hospitalization, the patient underwent a fasting test, during which the glucose infusion was stopped and the patient did not eat anything. The hypoglycemic response was achieved in approximately 5 h. Ketone levels were not measured. The levels of plasma insulin, blood glucose, and C-peptide during the fasting test (Table 1) excluded the presence of an insulinoma. Furthermore, she had low levels of IGF-1 and normal levels of IGF-2 (Table 1).

**Figure 1:**
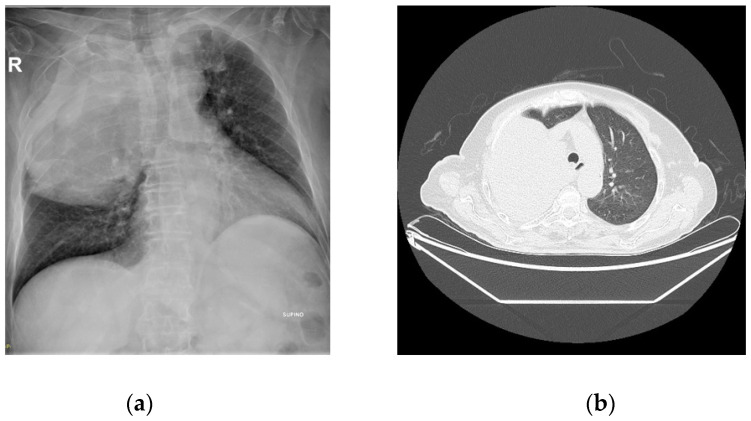
Radiographic images. (**a**) RX (X-Ray) and (**b**) CT scan show a large mass, with a maximum diameter of 15 cm, which almost entirely occupies the right lung and displaces the mediastinal structures contralaterally.

In our case, the IGF-2/IGF-1 ratio was 9.5, higher compared to the normal ratio, but lower than the critical value of 10, although very close to it. Because of the high suspicion of paraneoplastic hypoglycemia, we decided to re-examine the thoracic lesion by X-Ray (RX) and CT scan, which indicated further growth of the lung formation (Figure 1). Despite the large size of the thoracic mass, the patient had no respiratory symptoms. A new biopsy was performed, and it suggested the diagnosis of SFT.

**Figure 2:**
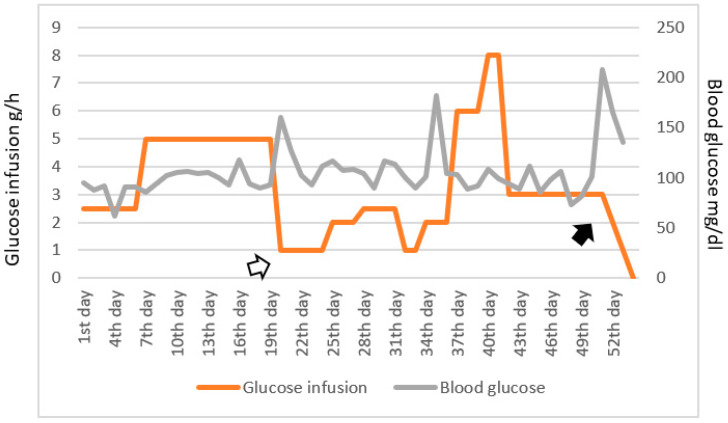
Time course of blood glucose levels and glucose infusion. Blood glucose levels (mg/dL) are represented in gray and glucose administration (expressed as hourly administration of glucose grams/h) is in orange. The white arrow represents steroid administration with relative reduction of glucose infusion; the black arrow indicates the surgical removal of the tumor with an evident increase in blood glucose levels.

The therapy used in the patient included both a correction of hypoglycemia and definitive cure of the neoplasm. To correct the constant hypoglycemia, a glucose solution was infused intravenously (iv), and its titration (infusion rate and concentration) was modulated according to clinical response. We also attempted to correct hypoglycemia using prednisone 25 mg/day: the clinical response was excellent with an important reduction in the amount of glucose administered. However, we had to interrupt this therapy on psychiatric recommendation, due to the occurrence of hallucinatory psychosis. This led to the need to increase iv glucose again (Figure 2).

**Figure 3:**
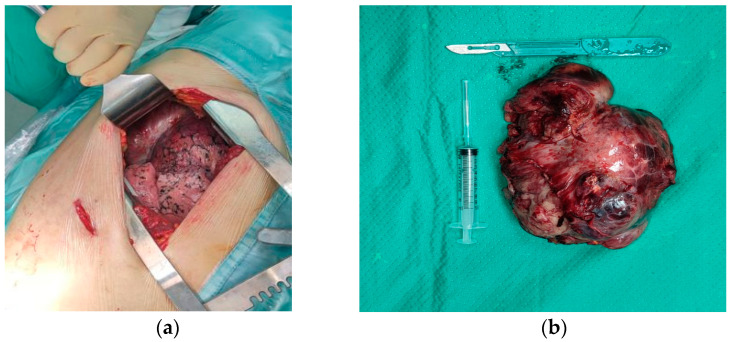
Surgical approach. (**a**) Thoracotomy with exposure of pleura and lung. (**b**) Excised tumor about 17 cm in the largest diameter.

A multidisciplinary team of endocrinologists, surgeons, anesthesiologists and cardiologists referred the patient to surgery. A thoracotomy was performed and a neoplasm measuring 17 cm was removed (Figure 3). Histology confirmed the diagnosis of SFT with a high risk of progression and metastasis.

After surgical removal, the patient’s blood glucose levels rose massively, so the glucose infusion was first reduced and then stopped (Figure 2). Since discontinuation, the patient did not have a new episode of hypoglycemia until discharge.

## 3. Discussion

Hypoglycemia in the patients without diabetes is a diagnostic challenge for the endocrinologist because it is a manifestation of several causes. The presence of refractory hypoglycemia should always prompt a search for the many causes of it, including rare causes such as NICTH and DPS.

The biochemical features of NICTH are low blood glucose with adequately suppressed insulin and C-peptide concentrations; GH levels are also low [7]. Furthermore, IGF-1 levels are usually below the normal range, while IGF-2 levels may be high, normal or low. In our case, the absolute levels of IGF-1 and IGF-2 were low and normal, respectively. These findings apparently excluded the diagnosis of DPS. However, IGF-2 levels alone are not representative of NICTH; in fact IGF-1 and IGF-2 levels alone can be confusing and lead to a wrong diagnosis. Therefore, the best marker for the diagnosis of NICTH is the assessment of the IGF-2/IGF-1 ratio [11]. The normal ratio is <3 and a ratio >10 is widely considered to be diagnostic of NICTH [7,12]. In our case, the IGF-2/IGF-1 ratio was high, with a value of 9.5, above the cut-off of normality of 3 and close to the critical value of 10. The high level of the ratio, although below the diagnostic cut-off, was suggestive of DPS in combination with the clinical history and pathological findings of SFT.

A multidisciplinary approach is often necessary. The principal treatment of DPS is surgical resection, which is curative for hypoglycemia when it is complete; however, cases of the resolution of hypoglycemia, even with subtotal resection, have been reported [12]. Large tumor size, metastasis, impairment of nearby structures, and clinical status of the patient represent some of the reasons against surgery. Several pharmacological options can be an option in these cases and in those in which hypoglycemia is severe enough to require bridging therapy or additional treatment. Recombinant human GH—at doses of 3–12 mg/day—has had some success in the treatment of hypoglycemia. The mechanisms of action are probably multifactorial, involving the counterregulatory action of GH and stimulating hepatic production of IGF-1, ALS, and IGFBP3 with a rise of ternary complex formation. Glucocorticoids are widely used in DPS. High-dose glucocorticoid therapy has an abrupt effect on symptomatic hypoglycemia. An increase in IGF-1 and a decrease in big-IGF-2 and total IGF-2 are the pharmacological mechanisms. Clinicians should consider combining the two therapies because it could guarantee greater success than monotherapy, with better control of side effects at lower doses [12]. Our first therapeutic approach was based on the need for metabolic stabilization and the avoidance of hypoglycemia. Initially, the use of oral steroid therapy drastically reduced the administration of glucose solution, achieving a good glycemic control. After a few days, due to the side effects, it was necessary to stop the prednisone therapy and to resort to a surgical approach, after which the hypoglycemia no longer occurred. It is important to note that due to the rarity of this syndrome, there is no information from randomized trials to provide strong evidence about the optimal treatment.

## 4. Conclusions

Although DPS and NICTH in general are rare, patients with recurrent hypoinsulinemic hypoglycemia should always be tested and screened for these conditions. Greater clinical attention and more widespread use of IGF-2 testing is needed to ensure correct diagnosis, although the availability of IGF-2 testing appears to be limited and contributes to the underdiagnosis of this condition. The role of the clinician is to achieve a definitive cure of the tumor. However, if the neoplasm is inoperable, the clinician must implement strategies to avoid the complications of hypoglycemia and ensure a good quality of life.

## Figures and Tables

**Table 1:** Blood tests results.

	Value	Normal Range
**Insulin autoantibodies**	1.92 IU/mL	<10
**IGF-1**	50.1 μg/L	90–360
**IGF-2**	476 μg/L	200–1000
**Fasting test**		
**Blood glucose**	46 mg/dL	50–100
**Plasma insulin**	<0.2 μU/mL	3.2–16.3
**C-peptide**	0.1 ng/mL	0.8–4.2

## Data Availability

No new data were created or analyzed in this study. Data sharing is not applicable to this article.
